# Exploring Microphone Technologies for Digital Auscultation Devices

**DOI:** 10.3390/mi14112092

**Published:** 2023-11-12

**Authors:** Matteo Zauli, Lorenzo Mistral Peppi, Luca Di Bonaventura, Valerio Antonio Arcobelli, Alberto Spadotto, Igor Diemberger, Valerio Coppola, Sabato Mellone, Luca De Marchi

**Affiliations:** 1 ARCES—Advanced Research Center on Electronic Systems for Information and Communication Technologies “Ercole De Castro”, University of Bologna, 40136 Bologna, Italy; matteo.zauli7@unibo.it (M.Z.); lorenzomistral.pepp2@unibo.it (L.M.P.); valerio.coppola3@unibo.it (V.C.); 2 STMicroelectronics, ARCES, 40136 Bologna, Italy; luca.di-bonaventura@st.com; 3 Department of Electrical, Electronic, and Information Engineering “Guglielmo Marconi”, University of Bologna, 40136 Bologna, Italy; valerio.arcobelli2@unibo.it (V.A.A.); sabato.mellone@unibo.it (S.M.); 4 Institute of Cardiology, Department of Medical and Surgical Sciences, University of Bologna, Policlinico S.Orsola-Malpighi, via Massarenti 9, 40138 Bologna, Italy; alberto.spadotto2@unibo.it (A.S.); igor.diemberger@unibo.it (I.D.); 5 UOC di Cardiologia, IRCCS Azienda Ospedaliero-Universitaria di Bologna, Dipartimento Cardio-Toraco-Vascolare, via Massarenti 9, 40138 Bologna, Italy

**Keywords:** digital stethoscope, auscultation, wearable, microphones, MEMS, piezoelectric, electret condenser, technologies comparison

## Abstract

The aim of this work is to present a preliminary study for the design of a digital auscultation system, i.e., a novel wearable device for patient chest auscultation and a digital stethoscope. The development and testing of the electronic stethoscope prototype is reported with an emphasis on the description and selection of sound transduction systems and analog electronic processing. The focus on various microphone technologies, such as micro-electro-mechanical systems (MEMSs), electret condensers, and piezoelectronic diaphragms, intends to emphasize the most suitable transducer for auscultation. In addition, we report on the design and development of a digital acquisition system for the human body for sound recording by using a modular device approach in order to fit the chosen analog and digital mics. Tests were performed on a designed phantom setup, and a qualitative comparison between the sounds recorded with the newly developed acquisition device and those recorded with two commercial digital stethoscopes is reported.

## 1. Introduction

The auscultation of a patient’s chest is a safe, non-invasive, and cost-effective diagnostic technique used by clinicians to diagnose various pulmonary and heart diseases. It is an important part of the patient examination and, even nowadays, is considered an essential part of a clinical diagnosis despite the broad range of available diagnostic technologies. Auscultation can be used to evaluate the airflow through the tracheobronchial tree by distinguishing normal respiratory sounds from abnormal ones (e.g., wheezes, pleural friction, rubs, and crackles) [1] or to assess heart sounds (valve opening and closure) or murmurs (blood flow turbulence) when looking for cardiovascular disorders [2].

The design of a digital auscultation device requires a careful evaluation of the sounds occurring in the chest, mainly the tones generated by the lungs and heart. The heart sounds are the result of blood flow, which produces several different sounds, usually referred to as S1, S2, S3, and S4 [2,3]. In a healthy patient, the most important heart sounds are S1 and S2: S1 is produced by mitral and tricuspid valve closure; S2 is due to aortic and pulmonic valve closure. Conversely, S3 is generated when a rapid reduction of blood supply from the left atrium to the ventricle occurs, and S4 is related to a failure of the heart in the diastolic period. In addition, murmurs generated by the whooshing made by rapid and turbulent blood flow can be acquired. Each of these sounds is characterized by specific frequency ranges: S1: 50–60 Hz, S2: 80–90 Hz, S3: 20–30 Hz, S4: <20 Hz, and murmurs: 200–600 Hz [4,5].

Lung sounds are produced during breathing. Similar to what happens for the heart beating, respiratory tones that are audible from the chest can be divided into normals and abnormals [6]. The normal sounds and relative frequency ranges are the following: bronchial: 60–700 Hz [7], and vesicular: 100–1000 Hz [6]. The abnormal noises and their frequency bands are [6] wheeze: 100–5000 Hz, stridor: >500 Hz, rhounchus: around 150 Hz, fine crackle: around 650 Hz, coarse crackle: about 350 Hz, pleural friction: rub < 350 Hz, and squawk: 200–300 Hz.

In this work, a signal conditioning circuit for analog microphones tuned to chest sound frequencies is reported. The focus will be precisely on choosing the best microphone technology for the auscultation practice. In the literature, there are many examples of microphone technologies being compared [8,9] in a large number of applications, including human body auscultation [10,11]. Nevertheless, the work on contact mics is quite old [10,12], and these works do not take advantage of the advancements in MEMS mic manufacturing technologies [11]. Regarding the practical usage of MEMS microphones in digital auscultation, some preliminary evaluations have been conducted [13,14], and a few auscultation devices have been presented [15,16], but there is a lack of comparison studies among the different microphone technologies.

Indeed, four mic manufacturing technologies are comparatively evaluated in this work: thin-disk piezoelectric diaphragms [17], electret condensers [18], an analog MEMS [19], and a digital MEMS [19]. These electroacoustic transducers are pivotal in enabling the design and development of electronic stethoscopes [20,21,22].

The introduction of digital stethoscopes has garnered significant attention from researchers [22,23,24], and products are available on the market [25,26]; these new devices seem to be not yet widely adopted for biomedical purposes regardless of many cases of validation [21,27,28]. Besides, digital stethoscopes have superior performance in terms of wrt than acoustic ones in patient diagnosis because of the ability to record and visualize, as well as to condition and amplify, the signal giving acoustic feedback, which is not audible when using classical methods [16,20]. The aim of our article is to contribute to the promotion of digital auscultation systems, emphasizing the integration of new technologies like MEMSs and providing a foundation for the development of compact, long-term-use devices in this field.

In this context, the intent of this work is to present a technology comparison, including MEMS solutions. Auscultation device testing is still in its infancy, and no standard is available [29], yet a possible approach consists of using phantoms that are either air-coupled [30] or gel-based [31]. In this work, we adopted a solution similar to the one proposed in [32], based on a water-filled polymer ball.

The tests focused on frequencies up to 5 KHz since the coverage of all the chest sounds described above requires a frequency band in the range of 1–5 KHz. The audio benchmarks taken into account for each microphone are the frequency range, signal-to-noise ratio (SNR), and the average power in the 1–5 KHz frequency range.

The paper is organized as follows. In Section 2.1, the microphones and the gold standard selection are reported, with a technical description for each device. Furthermore, in the same Section, the design and development of the housing of the mics (chestpiece), the acquisition system circuitry, and the testing of the various transducers on a purposely built setup are described. Section 3 presents the microphone comparison results. Conclusions complete the paper.

## 2. Materials and Methods

### 2.1. Microphones

The microphone is an electroacoustic transducer that converts sound waves into an electrical signal. A mechanical system set into oscillations by sound fields is in charge of the conversion, usually via a diaphragm. The diaphragm vibrates when it is hit by acoustic energy; this vibration is then converted into an electrical signal by different kinds of electromechanical coupling solutions.

Microphones are categorized by their transducer technology (for example, dynamic, condenser, etc.) and by several characteristics, such as those listed in Table 1: SNR, sensitivity and acoustic overload point (AOP), and frequency response. The SNR is calculated with a reference acoustic level of 1 Pascal, which is standardized as a 94 dB sound pressure level (SPL) 1 kHz tone at the microphone’s capsule. The same acoustic reference is used to evaluate sensitivity as well, which is the microphone signal output magnitude when its capsule is exposed to a standard pressure level. The AOP is the SPL value when the harmonic distortion present in a signal is equal to 10%. If the SPL value is higher than the AOP, the harmonic distortion will be higher than 10%. The main features are shown in Table 1 to provide an overview of the four chosen microphones, and more details are provided in the following sections. The reported list is the result of a careful selection of the best-in-class sensors, with the focus being not only on performance but also on the constructive approach, size, and cost in terms of addressing auscultation. Therefore, we have selected Murata’s piezoelectric diaphragms, two STMicroelectronics MEMS microphones (one analog and the other digital), and a high-quality electret condenser mic.

It is worth noting that, in Table 1, the SNR, sensitivity, and AOP values for the mic “7BB-35-3L0” are missing because this component is used for general applications (vibration monitoring, sound detection, buzzer, haptic feedback, etc.), both as a sensor and as an actuator, so it is not specifically characterized for audio applications. More details are in the following subsections.

#### 2.1.1. Piezoelectric Diaphragms 7BB-35-3L0

**Figure 1:**
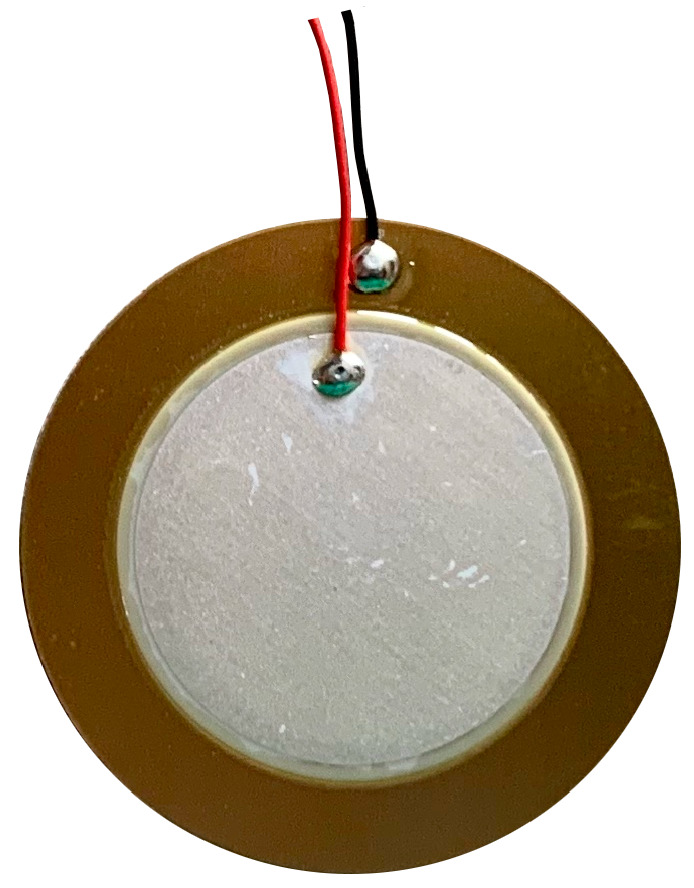
Murata piezoelectric diaphragm: 7BB-35-3L0.

The Murata “7BB-35-3L0” (Figure 1) is a lightweight, ultra-thin ( 0.53 mm), and circular piezoelectric diaphragm. It is based on the piezoelectric effect, and it is widely used in applications where contact is a requirement. Such types of sensors have already been applied in the detection of murmurs [38].

#### 2.1.2. Electret Condenser Primo EM272Z1

**Figure 2:**
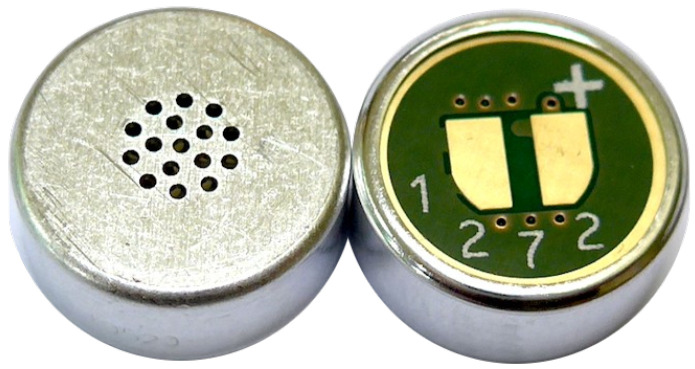
Primo electret condenser microphone: EM272Z1.

The EM272Z1 (Figure 2) is an omnidirectional microphone capsule with solder pads for two wires connection. It is based on a field effect transistor and an electret (crasis of ‘electricity’ and ‘magnet’), a dielectric material with a permanent dipole polarisation removing the need for a polarizing power supply. This microphone is characterized by an SNR of 80 dB, −28 ± 3 dB, and a maximum input sound pressure level of 119 dB. The manufacturer recommends their mic for recording natural sounds, backgrounds, and ambiance, thanks to the low noise and high sensitivity characteristics.

#### 2.1.3. Analog MEMS MP23ABS1

**Figure 3:**
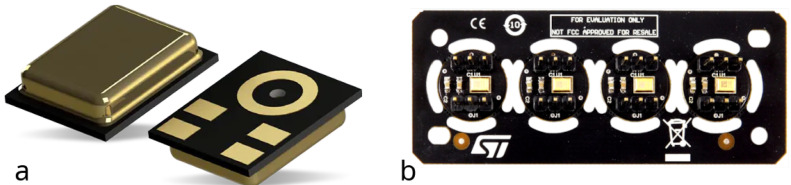
STMicroelectronics analog MEMS microphone: MP23ABS1: (**a**) device; (**b**) STEVAL–MIC004V1 evaluation board.

The MP23ABS1 (Figure 3a) by STMicroelectronics is a low-power (maximum 150 μA) and compact device (3.5 × 2.65 × 0.98 mm) with a 64 dB SNR, an omnidirectional sensitivity of −38 ± 1 dBV (at 94 dBSPL, 1 KHz), a 130 dBSPL AOP, and a flat frequency response of up to 15 KHz. This device comes with a metal casing to improve product robustness and reliability. Moreover, the substrate embeds capacitance plating to enhance RF immunity, and the base is provided with a sound pickup hole. Applications for this microphone include wearables, hearables, and smart speakers.

The testing of the MP23ABS1 mic was performed using an evaluation board, namely the STEVAL–MIC004V1 (Figure 3b). It is a daughterboard containing four single–coupon boards, hosting each one an MP23ABS1 and a double, three-pin header (2.54 mm pitch).

#### 2.1.4. Digital MEMS IMP34DT05

**Figure 4:**
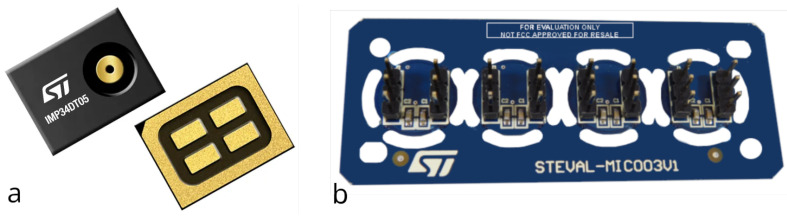
STMicroelectronics digital MEMS microphone: IMP34DT05: (**a**) device; (**b**) STEVAL–MIC003V1 evaluation board.

In the STMicroelectronics product portfolio, there are also digital outputs. The IMP34DT05 (Figure 4a) is a digital microphone that includes a small-form factor (3 × 4 × 1 mm), which is a dedicated circuit to provide a digital signal encoded with pulse density modulation (PDM). In addition, it offers a low-power consumption (650 μA), omnidirectional directivity, a 64 dB signal-to-noise ratio, −26 ± 3 dBFS of sensitivity, and an AOP of 122.5 dBSPL. The device’s package is made of plastic with a ground ring around the sound pickup hole to enhance protection against electrostatic discharges.

Some recommended applications are sound detection, noise canceling, predictive maintenance, and vibration monitoring.

For testing the IMP34DT05, we have, again, used the STEVAL–MIC003V1, which features four coupon boards for easy interfacing with the digital microphone on our acquisition board.

### 2.2. Gold Standard

For the selection of the transducer most suited for chest auscultation, a gold standard benchmark is needed. However, it is important to note that, due to industrial confidentiality, the specific technical details of these devices are not publicly available. Nevertheless, we have chosen two of the most commonly used digital stethoscopes: Thinklabs One and eKuore. These were used in the test setup (Section 2.5) alongside the selected microphones, enabling a performance-based comparison in terms of the sensitive frequency band and a floor noise analysis.

#### 2.2.1. Thinklabs One

**Figure 5:**
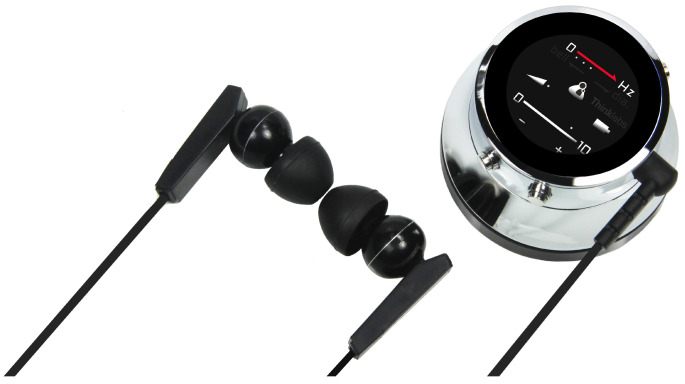
Thinklabs One; digital stethoscope.

Thinklabs One [26] (Figure 5) is a digital stethoscope that features multiple filter choices and can be used to acquire sounds from the heart, lungs, and other parts of the body. Unlike traditional devices, the electronic amplification of sound is regulated through volume control. The volume and filtering controls can be used via buttons located on the device itself. The user can auscultate body sounds via external earphones or record via a device with an onboard device (laptop, smartphone, etc.) connected via a 3.5 mm audio jack to the device.

Briefly, here are some of the device’s characteristics:

- Audio transmission: 3.5 mm audio jack;
- Audio amplification: 40 dB;
- Audio filters: Five selectable bandpass filters;
- Display: Volume, filter, battery;
- Power supply: 5 V DC (compatible for USB charging)—internal lithium-ion battery;
- Battery life: 4 h;
- Dimensions: 46 mm × 28 mm;
- Weight: 50 g.

For the bandpass filters of About One, in our test, we used the wideband mode filter. The filters are

1. 30–500 Hz: heart sounds, especially S3;
2. 60–500 Hz: heart sounds, whether filter 1 is too intense;
3. 80–500 Hz: lung sounds and heart valve clicks, S2 splits;
4. 100–1000 Hz: lung sounds;
5. 20–2000 Hz: wideband mode.

#### 2.2.2. eKuore

**Figure 6:**
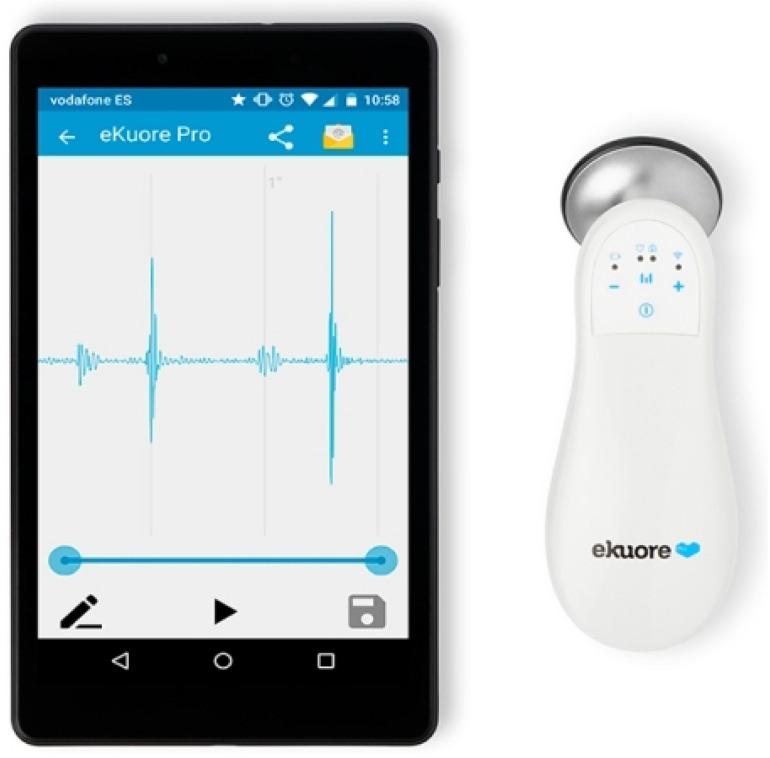
eKuore, digital stethoscope.

eKuore (Figure 6) is a stethoscope that exploits a Wi-Fi connection to communicate with any smartphone or tablet via a free dedicated app. On those devices, the phono-cardiograms of the heart and lungs can be recorded and viewed in real time or shared with remote users. In addition, the auscultations can be performed using a headphone set, thanks to the device’s 3.5 mm jack connector.

Features:

- Audio transmission: Wireless IEEE 802.11b/g and 3.5 mm audio jack;
- Audio amplification: 10 dB;
- Audio filters: three selectable bandpass filters;
- Display: Volume, filter, and battery;
- Power supply: 5 V DC (compatible for USB charging)—internal lithium-ion battery;
- Battery life: 7 h;
- Dimensions: 130 mm × 50 mm × 30 mm (W × D × H);
- Weight: 85 g.

Concerning the onboard filters, for the test, we used the eKuore widest band filter, as we did for the Thinklabs One stethoscope. The three filters are

1. 50–150 Hz: cardiac mode;
2. 50–500 Hz: lung mode;
3. 40–600 Hz: wide mode.

### 2.3. Chestpiece

**Figure 7:**
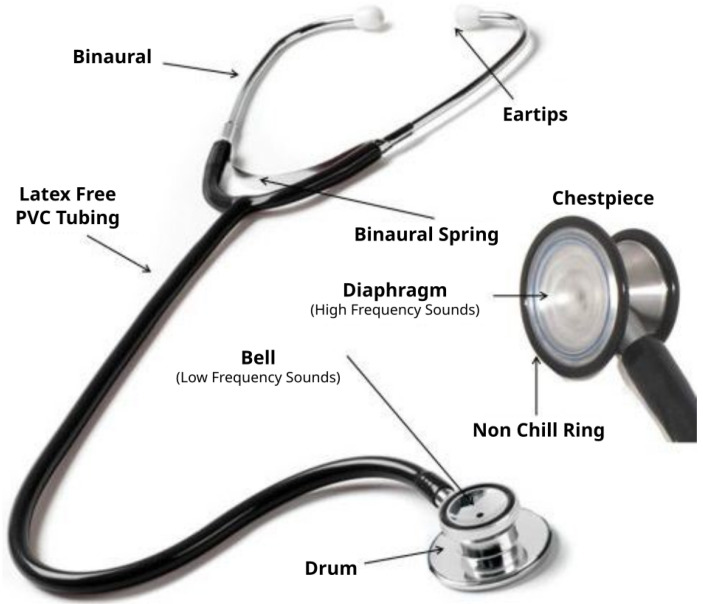
Stethoscope parts [40].

An important element of the stethoscope (illustrated in Figure 7) is the chestpiece. For this reason, for the characterization of the device’s performance, we considered how this component might affect the results in terms of evaluating the different solutions to house the mics, following the approach reported in [39]. More specifically, a chestpiece consists of two parts: a front side and a back side (see Figure 7), usually referred to as the bell and diaphragm. The diaphragm may have a large diameter and is especially relevant for high-frequency sounds, such as those produced by breathing. The bell, known for its smaller size, is suitable for capturing low-frequency sounds, such as heart murmurs or pediatric respiratory sounds. Usually, the diaphragm is made by a base metal drum or cup and by a rigid, flat, and thin plastic disc of bakelite or an epoxy–fiberglass compound, whereas the bell is a simple metal elongated cup. The noises generated by the human body cause the plastic disc to vibrate (when it is in contact with the patient’s skin), which then generates the sounds heard when using the stethoscope chestpiece.

**Figure 8:**
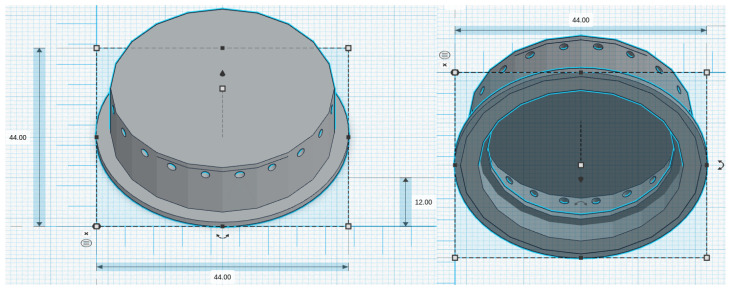
Cup for piezoelectric diaphragm.

**Figure 9:**
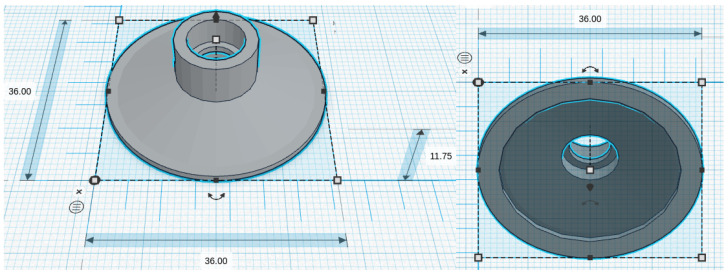
Cup size: small, for electret condenser mic.

**Figure 10:**
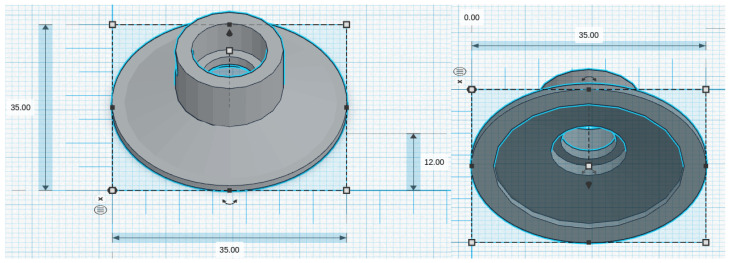
Cup size: small, for MEMS mics.

**Figure 11:**
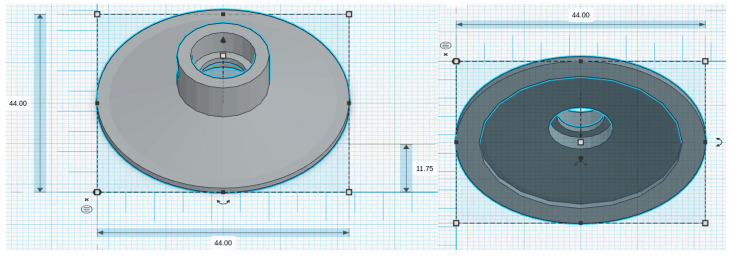
Cup size: big, for electret condenser mic.

**Figure 12:**
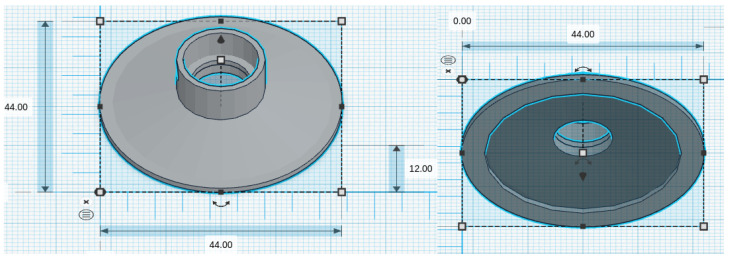
Cup size: big, for MEMS mics.

We considered five chestpiece models for the four microphones: for the piezoelectric diaphragm, a single-size drum (Figure 8, inspired by [39]) was realized; for the electret condenser and MEMS solutions, two chestpieces were developed (based on stethoscope’s chestpiece parts and [39]), with the difference being the small (Figure 9 and Figure 10) or large (Figure 11 and Figure 12) dimensions. The piezo diaphragm size was fixed, so it was not possible to have two different cups for it, whereas, for the other solutions, two different cup sizes were analyzed in order to test the different frequency responses, akin to the bell and diaphragm parts of the chestpiece.

All the cups were 3D-printed with a PLA (polylactic acid) plastic filament. In the following pictures, the designs obtained using the web app for 3D design Thinkercad [41] are depicted. The cups’ dimensions are suitable to host the diaphragm plastic disc. More specifically, the “3M™ Littmann® Stethoscope Spare Parts Kit Classic III™ Cardiology IV™ and CORE, 40,016, Black, 10 Kit/Case” [42] was purchased, a kit with two plastic diaphragms suitable for the various cups and for the large and small sizes. Regarding the insertion of the respective sensor inside the cups, a circular constriction can be observed. For the piezoelectric solution, this constriction is visible only from the bottom view, approximately halfway up the chamber. For the other solutions, it can be noticed from both the top and bottom views at the beginning of the conical chamber. The sensor is placed and attached at this point to ensure an airtight seal, preventing any air leaks and allowing the full pressure of the air chamber below to act on the microphone sensor.

It is worth noting that plastic was selected for the cups in this study. While metal is theoretically superior in terms of sound conduction properties, it is noteworthy that plastic is also a material commonly employed in professional stethoscopes, both acoustic and digital. The choice of plastic aligns with the goal of exploring possibilities for wearable systems. Wearable devices prioritize characteristics like comfort, lightness, and miniaturization, which can be more challenging to achieve with metal construction.

### 2.4. Electronic Prototype

**Figure 13:**
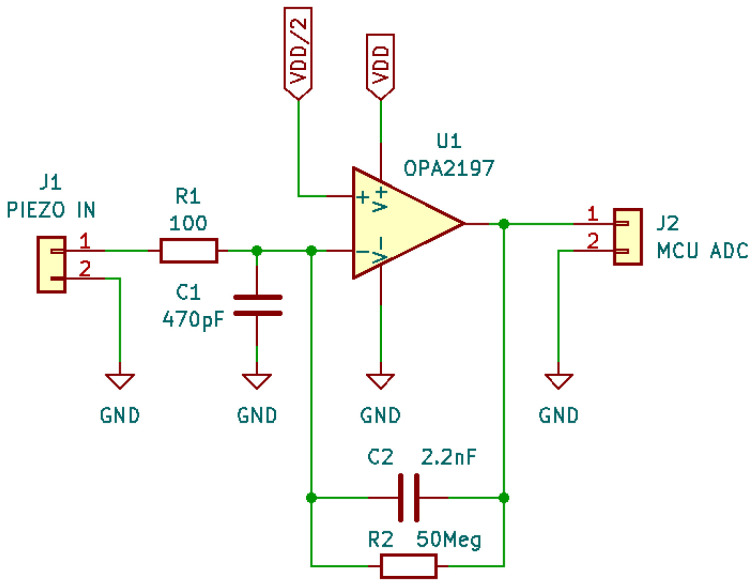
“Piezoelectric preamplifier board” electronic schematic.

**Figure 14:**
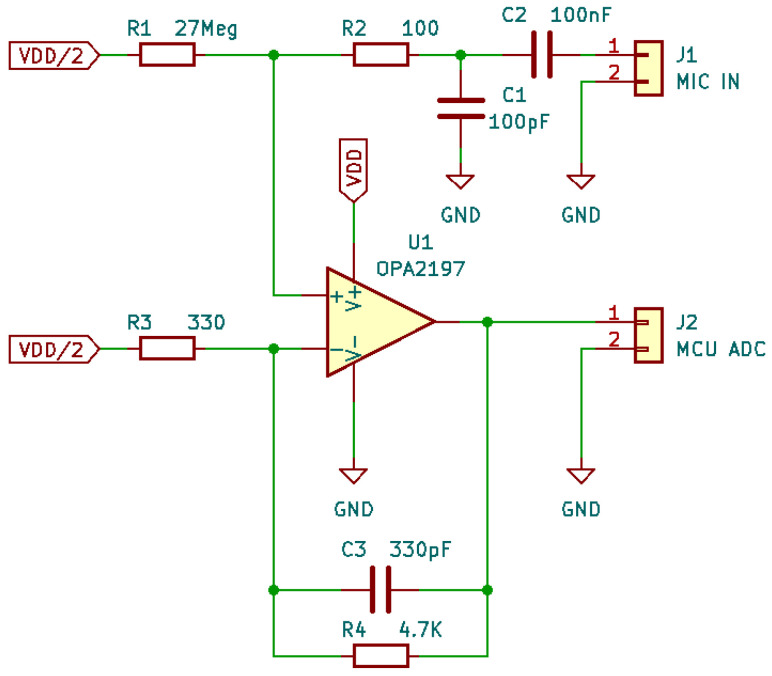
“Analog preamplifier board” electronic schematic.

**Figure 15:**
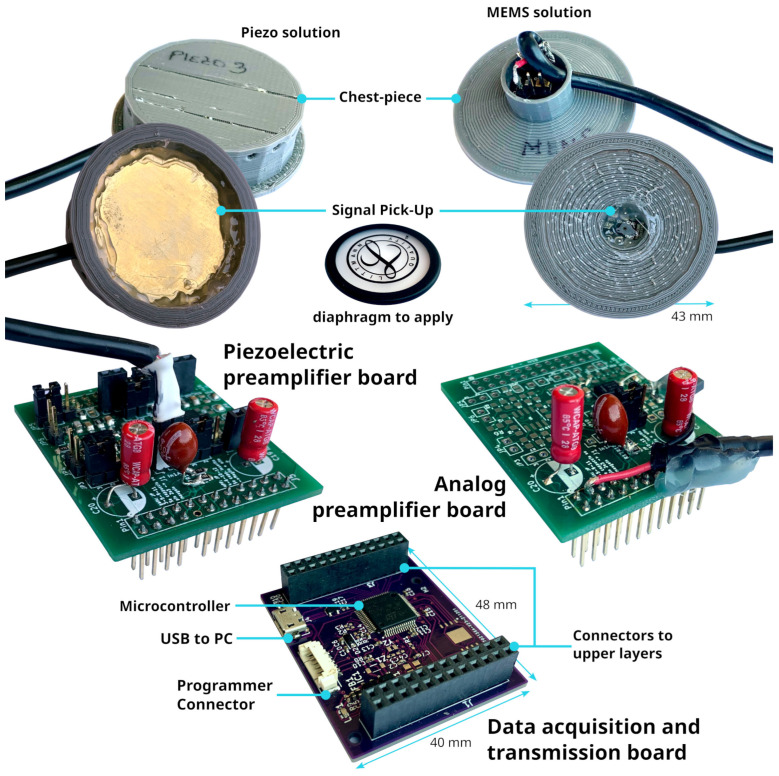
The developed auscultation device prototype components.

The aim of this research is to compare microphone technologies suitable for patient auscultation. Since the target of these technologies is intended for electronic stethoscope applications, we have developed a digital acquisition system to evaluate the mics using a plausible acquisition chain. The electronic prototype has been developed as a modular system to support both digital and analog microphones and a dedicated board for signal measurement and transmission. In particular, the following components have been used:

- The “Data acquisition and transmission board” hosts the Microcontroller Unit for data acquisition features and acts as a USB PC peripheral for data transfer to a personal computer for data postprocessing.
  The main component, an STM32 MCU, is a high-performance STM32L552RE, which integrates an ARM Cortex-M33 core operating at a frequency of up to 110 MHz, with a single-precision floating point unit, a digital signal processing instruction set, 256 KiB of SRAM, and 512 KiB of flash memory. It embeds two fast ( 5 Mbps) 12-bit analog-to-digital converters (ADC), four digital filters for external sigma delta modulators (a digital filter for a sigma-delta modulator peripheral—DFSDM), and various communication interfaces: full-speed USB, SPI, UART, etc.
- The “Piezoelectric preamplifier board” is the interface for piezoelectric diaphragms; it embeds two precision dual-channel operational amplifiers (op-amp) in an inverted configuration, with an overall gain of around 22 dB in around the 1 Hz- 20 KHz band and a noise voltage level of 12 μV.
  The op-amps are from a Texas Instrument, the OPA2197 [43], which is a low-noise ( 5.5 nV/Hz at 1 KHz) and wide-bandwidth ( 10 MHz) dual-channel Op-amp, with a low-offset voltage of ±100 μV and a low-bias current of ±5 pA. The electronic schematic can be found in Figure 13;
- The “Analog preamplifier board” is suitable for MEMSs and condenser analog microphones.
  As the “Piezoelectric preamplifier board”, it is based on OPA2197 but with a different configuration: a non-inverting circuit with resistive feedback. The gain is around 23 dB in a frequency band of 1 Hz–20 KHz. The schematic is shown in Figure 14;

In Figure 15, the printed circuit boards of the above-mentioned components are depicted, together with a top and bottom view of a couple of chestpieces that were realized to test the various microphones discussed in Section 2.1, and the Littmann^®^ diaphragm plastic disc introduced in Section 2.3. Moreover, it is worth noting that a flexible stacking architecture was implemented so that the “Data acquisition and transmission board” could host both the “Analog preamplifier board” and “Piezoelectric preamplifier board” to interface the MCU ADC channels with the analog microphones and piezoelectric solution. The digital mic can be connected directly to the MCU DFSDM peripheral by employing the “connectors to upper layer” without any additional board or adapter. A programmer connector for code loading and debugging purposes is included in the data acquisition board, together with a USB port to use the device as a serial PC peripheral. Currently, the device is not used as a standalone system but rather as a test bench for mic characterization. Therefore, it is connected via USB to a laptop, and it is controlled by a MATLAB^®^ 2021b interface–script; the acquisition starts when a “Start” command is received by the MCU Layer. A multi-thread buffer strategy allows data to be sent to the host by continuously recording signals without any data loss. Similarly, the acquisition ends when the “Stop” command is received by the MCU Layer. At this stage, the device is powered by the laptop battery itself. The MATLAB software saves the audio signals and transmits them to the audio output of the PC for real-time listening. In order to perform high-quality acquisition, the system features an enhanced dynamic range to achieve a best-in-class digital conversion with a sampling frequency of 44 Ksample/second/channel at a 12 bit resolution for the analog signals. For the digital mic, the MCU DFSDM interface performs sampling at a frequency of 44 Ksample/second/channel at 16 bits. The various board configurations have the capability to acquire up to four different channels simultaneously.

The main features are listed here:

- Audio transmission: Full–speed USB;
- Audio amplification: 22–23 dB;
- Audio filters: Custom on MATLAB;
- Power supply: 5 V DC;
- Dimensions: 40 mm × 48 mm × 25 mm (W × D × H).

### 2.5. Setup

**Figure 16:**
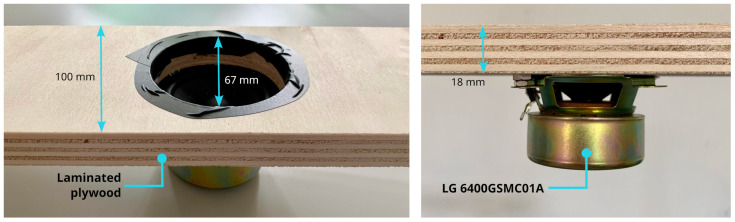
Laminated plywood board; part of the auscultation phantom setup to test the microphone technologies.

**Figure 17:**
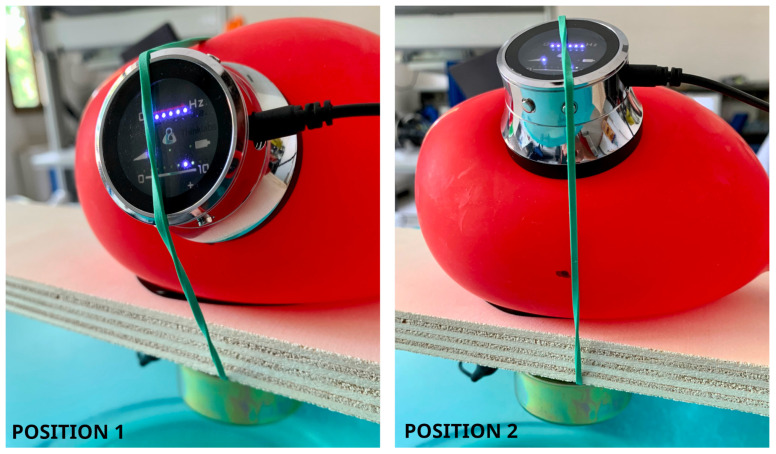
Auscultation phantom setup: on the left side, Position 1; on the right, Position 2.

**Figure 18:**
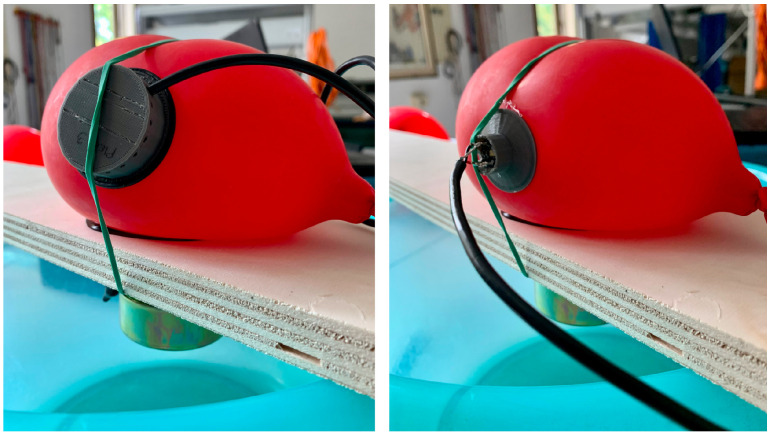
Test of piezoelectric diaphragm (left) and digital MEMS (right) in Position 1.

It is worth noting that even if many experimental setups have been proposed in the literature [31,44], no standard procedure to characterize auscultation device performance has been developed [32,45]. In this work, the tests of the various microphones introduced in Section 2.1 were performed, with the setup illustrated in Figure 16, Figure 17 and Figure 18. In particular, the setup is made up of a laminated plywood board, on which a full range loudspeaker LG 6400GSMC01A (Figure 16—70 mm diameter, 25 W, 6 ohm impedance) and a latex rubber balloon filled with water are housed. The transducers were placed in different measurement positions, i.e., Positions 1 and 2 in Figure 17, in order to verify whether the acquisition performances are influenced by the wave direction of arrival. In detail, the balloon is a standard 26 cm party balloon, and its full water weight is 1.9 Kg. The green elastic band that holds the microphones in their position (see Figure 16 and Figure 17) is a standard No. 33 [46] band size ( 90 mm × 3.0 mm). As mentioned in the introduction, this setup was inspired by [32]. More precisely, the dimensions of the plastic balloon were determined by the need to effectively cover the source ( 67 mm in diameter, as shown in Figure 16) and disperse sound within it.

The test bench was designed to mimic sound propagation in a medium with a human-body-like density (around 985 Kg/m^3^). For this reason, the balloon was filled with water (density equal to 997 Kg/m^3^). In the following, the list of mics and the related acquisition board configurations are provided:

- Piezoelectric Diaphragm 7BB-35-3L0: “Data acquisition and transmission board” and “Piezoelectric preamplifier board”, with firmware for the ADC input.
- Electret Condenser Primo EM272Z1 and analog MEMS MP23ABS1: “Data acquisition and transmission board” and “Analog preamplifier board”, with firmware for the ADC input.
- Digital MEMS IMP34DT05: “Data acquisition and transmission board”, with firmware for DFSDM.

**Figure 19:**
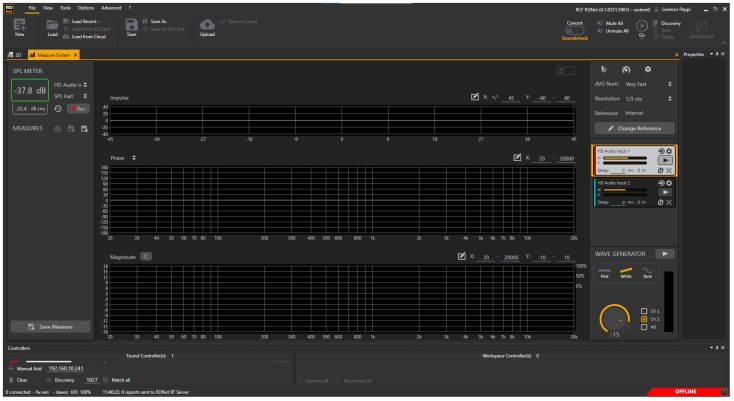
RDNet settings for white noise generation.

The two measurement positions for where we applied the microphones to the balloon are marked by a black marker, and for each one, we performed two measures: one generated by loudspeaker white noise and the other in total silence. The white noise was generated by connecting the setup speaker to a PC sound card featuring “RDNet” 4 software by RCF [47]. In Figure 19, the setting of RDNet can be seen. The purpose of the silent mode test is mainly to evaluate noise rejection in addition to operational bandwidth, whereas the white noise mode test focuses on evaluating the frequency response of the whole system.

## 3. Results

As anticipated, chest sound components are generated by different sources in different bands. The following list summarizes the frequency bands of interest, the details of which are provided in the introduction:

- Band 1: 8–100 Hz: related to mechanical cardiac events and containing information about valve functionalities and cardiac muscle contractility [48].
- Band 2: 20–1000 Hz: related to the opening and closing of the heart valves, along with the pumping of blood into the arteries and veins [49].
- Band 3: 20–200 Hz: heart sounds [49].
- Band 4: <100 Hz: seismocardiography, defined as the micromovements of the chest wall, in response to the pumping of blood with every heartbeat [50].
- Band 5: 60–5000 Hz: respiratory sounds [6,7].

In order to assess whether a device is capable of functioning in both cardiac and respiratory frequency ranges, the combined bands of interest span from 8 Hz to 5000 Hz. In Section 2.1, we outlined the microphone specifications, including SPL, AOL, and SNR, which are inherent characteristics. However, the primary objective of this article is to provide a comprehensive comparison of various technologies tailored specifically for contact-based auscultation applications. Consequently, we assessed parameters, such as frequency response and SNR, relevant to microphone usage in contact scenarios, as detailed below. We opted not to delve into the time domain due to the nature of the sounds used during testing (refer to Section 2.5). Moreover, a substantial aspect of this evaluation is already covered by the measured SNR, especially within the digital system employed for testing. Consequently, the performance in the time domain is reliant on the processing applied, which includes the utilization of adaptive filters as an illustrative example. Accordingly, a power spectral density (PSD) up to 10 KHz was analyzed for each microphone. More specifically, each signal (*x*) has been processed over three steps: (i) a calculation of the root mean square (RMS) value (Equation (1)), (ii) normalization (Equation (2)), and (iii) PSD estimation using the Welch periodogram (Equation (3)). In step (iii), the PSD was estimated with the longest possible segmentation to achieve high-frequency resolution: the signal was subdivided into eight segments with 50% overlap, and each one was windowed with Hamming windows. In Equation (3), the *m*th w(m) is the Hamming window of the *m*-th segment, *R* is defined as the window hop size, and *K* denotes the number of available frames (8 by “pwelch” MATLAB function). Then, the PSD signal is just an average of ${\hat{x}}_{m}$ periodograms across time.

(1) $$RMS=\sqrt{\frac{1}{N}\sum_{i=1}^{N}|x_{i}{|}^{2}}$$

(2) $$\hat{x}=\frac{x}{RMS}$$

(3) $$\begin{array}{cc} {\hat{x}}_{m}\left(n\right)=w\left(m\right)x\left(n+mR\right),n & =0,1,\ldots ,M-1,m=0,1,\ldots ,K-1,K=8\\ {\hat{S}}_{\hat{x}}^{W}\left(\omega_{k}\right) & =\frac{1}{K}\sum_{m=0}^{K-1}P_{{\hat{x}}_{m},M}\left(\omega_{k}\right) \end{array}$$

**Figure 20:**
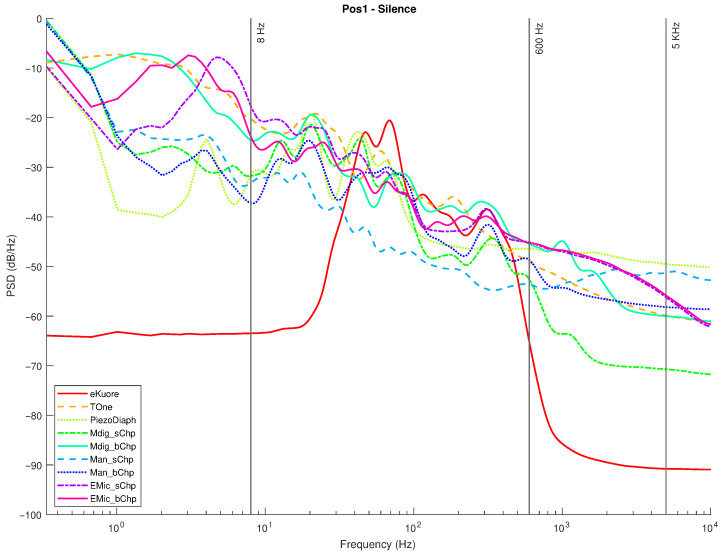
Position 1: normalized PSD microphone acquisitions in total silence.

**Figure 21:**
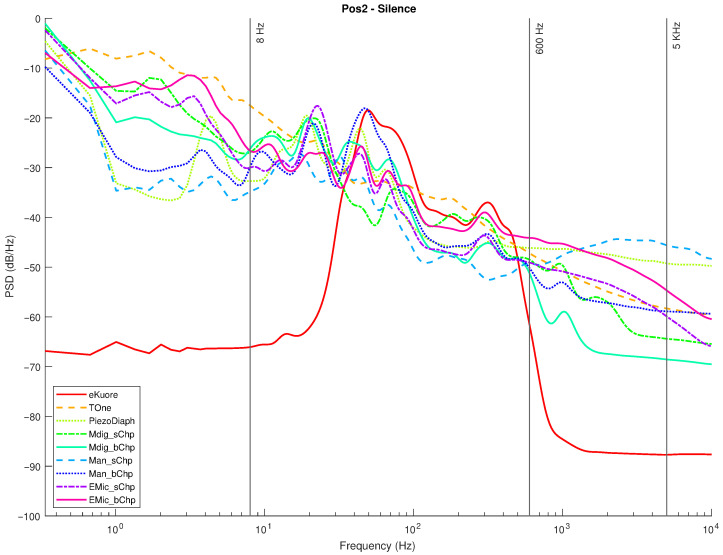
Position 2, normalized PSD microphone acquisitions in total silence.

**Figure 22:**
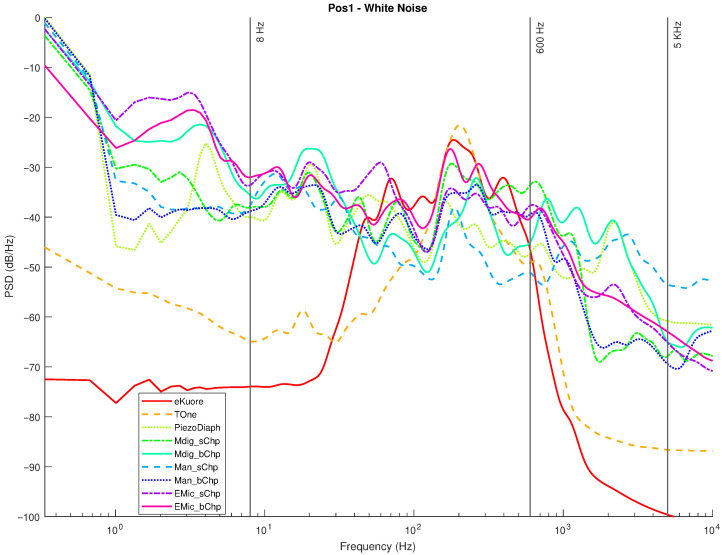
Position 1: normalized PSD microphone acquisitions with white noise.

**Figure 23:**
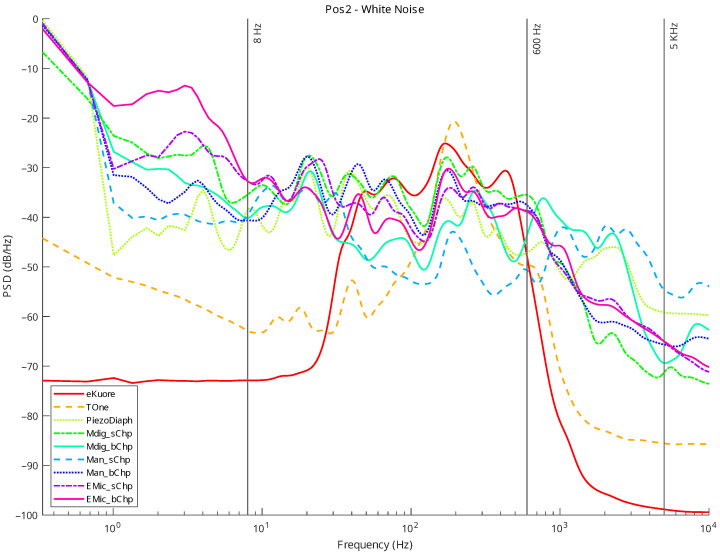
Position 2: normalized PSD microphone acquisitions with white noise.

In the following graphs, the results for the selected transducers (Section 2.1) and the gold standards (Section 2.2) are presented for Position 1 and Position 2. For each position, measurements in the silence mode (Figure 20 and Figure 21) and white noise loudspeaker playback mode (Figure 22 and Figure 23) were performed. Furthermore, the spectral coefficients were filtered with the 1/3-Octave smoothing filter [51,52], as is typically carried out for audio PSD graphing improvement.

In the plots, the spectrum for each mic or commercial stethoscope is displayed, labeled as

- *“EMic_bChp”*: Electret Condenser EM272Z1, with chestpiece size “big”;
- *“EMic_sChp”*: Electret Condenser EM272Z1, with chestpiece size “small”;
- *“Man_bChp”*: analog MEMS MP23ABS1, with chestpiece size “big”;
- *“Man_sChp”*: analog MEMS MP23ABS1, with chestpiece size “small”;
- *“Mdig_bChp”*: digital MEMS IMP34DT05, with chestpiece size “big”;
- *“Mdig_sChp”*: digital MEMS IMP34DT05, with chestpiece size “small”;
- *“PiezoDiaph”*: Piezoelectric Diaphragms 7BB-35-3L0;
- *“TOne”*: gold standard digital stethoscope: Thinklabs One;
- *“eKuore”*: gold standard digital stethoscope: eKuore.

From the PSD graphs, the effect of the onboard bandpass filter in the two commercial solutions, Thinklabs One and eKuore, is noticeable. In the context of white noise data acquisition, specifically in the plots depicted in Figure 22 and Figure 23, it is possible to provide a qualitative assessment of the transducers’ bandwidth. The key criterion for this evaluation involves using a reference threshold of −50 dB/Hz as the point where distinguishing the signal from the background noise becomes challenging. Based on these considerations, the best solutions in terms of bandwidth were identified as the analog MEMS with a small cup, the digital MEMS solution with a big cup, and the piezoelectric diaphragm. Table 2 summarizes the results of the upper graphs in terms of the upper-cutoff frequency at −50 dB/Hz. It is worth noting that the two commercial solutions exhibit a narrower bandwidth. However, this is influenced by the onboard processing they already implement, a feature not present in the other solutions under evaluation, where only the sensor’s performance is being assessed. In contrast, the gold standard solutions employ filtering techniques to enhance features such as SNR, as illustrated in the subsequent analysis. Nonetheless, an intriguing aspect of this study lies in considering higher bandwidths to capture a broader range of sounds, aligning with the specific frequency ranges mentioned in the introduction and revisited at the beginning of this section.

In order to provide a more accurate assessment of the operating frequency band, we calculated the average power for PSDs between 8 and 5000 Hz (see the discussion on the frequency band of interest in the Introduction and at the beginning of section Section 3). Then, the SNR was calculated in order to assess the noise rejection capabilities of each transducer on the frequency band mentioned above. The average power ($POW_{avg}$) is computed by integrating the PSDs using the rectangle method (Equation (4)), whereas the SNR values are the ratios between the $POW_{avg}$ (computed when white noise ($POW_{avg-wn}$ was applied) and the average power estimated during the silence mode tests ($POW_{avg-sil}$).

(4) $$POW_{avg}=\frac{1}{n}\sum_{i=1}^{n}\left(\omega_{i-1}-\omega_{i}\right)\left(\frac{{\hat{S}}_{\hat{x}}^{W}\left(\omega_{i-1}\right)+{\hat{S}}_{\hat{x}}^{W}\left(\omega_{i}\right)}{2}\right)$$

(5) $$SNR=10log_{10}\left(\frac{POW_{avg-wn}}{POW_{avg-sil}}\right)$$

The results of the above benchmark are reported in Table 3.

The gold standards exhibit superior performance compared to the selected technologies, primarily attributed to the advantages of onboard signal processing, which significantly enhance their overall performance. In contrast, our solution prioritized the comparison of raw data acquisitions among the microphone technologies. Within these technologies, it is worth noting that the digital MEMS application emerged as the best choice in terms of SNR. This observation excludes the gold standard and opens up the possibility of future improvements through the implementation of advanced filtering techniques and adaptive noise cancellation, leveraging the potential of a multichannel microphone system to bridge the performance gap. When looking ahead, it is important to consider how the integration of a multichannel microphone system in wearable devices is of interest, particularly in the context of noise cancellation. The currently presented hardware serves as a valuable testing platform and can be adapted to a wearable device while retaining the studied functionalities.

In summary, we have performed four audio recordings for each transducer by considering two different acquisition positions and two types of acquired sounds: white noise and complete silence. We selected two different acquisition locations to check for distinct sound propagation in the phantom setup. The two kinds of sounds were used: (i) a random signal with equal intensity at different frequencies reproducing the setup’s speaker of a white noise track and (ii) complete silence to compare the microphone sensitivities: The sounds recorded by the microphones and the designed acquisition system were postprocessed to extract multiple features: the PSD, PSD with octave filter, and the average power in the 8-5000 Hz band, from which the SNR can be calculated. Specifically, from the filtered PSD, the devices’ bandwidth can be estimated, while the SNR parameter illustrates noise immunity. From the achieved results, the best-performing technology is considered to be the digital MEMS-based technology, both for the large bandwidth (see Figure 22 and Figure 23 and Table 2) and the high SNR level (up to 15.99 dB; see Table 3).

In addition to the quantitative assessment reported so far, we conducted a preliminary qualitative assessment involving four trained cardiologists at the Department of Medical and Surgical Sciences of the University of Bologna. All physicians reported higher sound quality when using the digital MEMS microphone with respect to the other technologies.

## 4. Conclusions

This paper describes the suitability of different microphone technologies for chest auscultation. The selected transducers are based on various manufacturing approaches: piezoelectric diaphragm, electret condenser, analog MEMS, and digital MEMS. The performances of these solutions were compared to those achieved by using two commercial digital stethoscopes: Thinklabs One and eKuore. The comparison was based on the analysis of the parameters that are typically evaluated in studies related to auscultation sound acquisition methods. However, in this work, we did not focus only on testing the performance of the microphones but also on developing (i) an electronic digital acquisition device that could be used as a PC peripheral and (ii) purposely designed transducers, housing, i.e., the 3D-printed chestpieces. The aim was to understand how close the performances of both a low-cost, low-power device and the commercial devices (unsuited for prolonged use in wearable setups) are in terms of wrt. Finally, we realized an ad-hoc phantom setup to perform acoustic tests in simulating auscultation with appropriate sound playback, such as a white noise track. The results demonstrate how the MEMS technology can be reasonably considered for patient auscultation, particularly the digital MEMS-based microphone solution. Furthermore, the application of this technology opens up the possibility of auscultating sounds in a wider band, enabling more precise patient diagnosis by using low-cost devices.

## Figures and Tables

**Table 1:** Microphones.

Model	Manufacturer	Technology	Size [mm]	SNR [dBA]	Sensitivity	AOP [dB SPL]
7BB-35-3L0 [33]	Murata	Piezoelectric diaphragms	35 Ø	-	-	-
EM272Z1 [34]	Primo	Electret Condenser	10 Ø × 4.5	80 ^1^	−28 ±3 dBV ^2,3^	119
MP23ABS1 [35]	STM	Analog MEMS	3.5 × 2.65 × 0.98	64 ^1^	−38 ±1 dBV ^3^	130
IMP34DT05 [36]	STM	Digital MEMS	3 × 4 × 1	64 ^1^	−26 ±3 dBFS ^4^	122.5

^1^ A-weighted @ 1 KHz, 94 dB SPL.; ^2^0 dB = 1 V/Pa.; ^3^ @ 1 KHz, 94 dB SPL.; ^4^ Decibels, with respect to digital full scale; more info in [37].

**Table 2:** Upper-cutoff frequencies (Hz): Position 1 in the first row; Position 2 in the second row.

	EMic_bChp	EMic_sChp	Man_bChp	Man_sChp	Mdig_bChp	Mdig_sChp	PiezoDiaph	TOne	eKuore
**P1**	1198	1099	1118	3996	3008	1298	2937	705	633
**P2**	1192	1007	1074	4173	2808	1068	2886	663	618

**Table 3:** SNR evaluation (dB): Position 1 in the first row; Position 2 in the second row.

	EMic_bChp	EMic_sChp	Man_bChp	Man_sChp	Mdig_bChp	Mdig_sChp	PiezoDiaph	TOne	eKuore
**P1**	11.37	9.14	13.52	8.14	15.99	7.38	6.64	13.65	11.80
**P2**	9.14	4.21	4.18	4.17	9.67	7.64	7.01	16.10	11.92

## Data Availability

Data are contained within the article.

## Abbreviations

The following abbreviations are used in this manuscript:

MEMS	Micro-Electro-Mechanical Systems
SNR	Signal-to-Noise Ratio
AOP	Acoustic Overload Point
SPL	Sound Pressure Level
PDM	Pulse Density Modulation
ADC	Analog-to-Digital Converter
OP-AMP	Operational Amplifier
PSD	Power Spectral Density
RMS	Root Mean Square
